# Dietary Zinc and Fibre Source can Influence the Mineral and Antioxidant Status of Piglets

**DOI:** 10.3390/ani9080497

**Published:** 2019-07-29

**Authors:** Monika Holodova, Klaudia Cobanova, Zuzana Sefcikova, Marcin Barszcz, Anna Tuśnio, Marcin Taciak, Lubomira Gresakova

**Affiliations:** 1 Institute of Animal Physiology, Centre of Biosciences of the Slovak Academy of Sciences, Soltesovej 4-6, 04001 Kosice, Slovakia; 2 The Kielanowski Institute of Animal Physiology and Nutrition, Polish Academy of Sciences, Instytucka 3, 05-110 Jabłonna, Poland

**Keywords:** zinc glycine chelate, potato fibre, pigs, trace elements, zinc transporters

## Abstract

**Simple Summary:**

The post-weaning period is one of the most critical phases in a pig’s life, when trace mineral requirements increase because of the inadequate absorptive capacity of the intestine and reduced feed intake. Mineral bioavailability can be increased by using more available feed additives or the reduction of dietary antinutrients, such as phytates or fibres, in pig nutrition. Therefore, our study was focused on improving the absorption of trace elements, mainly zinc, from the small intestine of piglets using more efficient dietary sources, such as an organic form of zinc and potato fibre.

**Abstract:**

The study investigated the effect of dietary zinc glycine chelate and potato fibre on the absorption and utilisation of Zn, Cu, Fe, and Mn; the activity of Zn-containing enzymes (superoxide dismutase, SOD; alkaline phosphatase, ALP); and zinc transporter concentrations (metalothionein1, MT1; zinc transporter1, ZnT1) in tissues, with a special emphasis on the small intestine. Twenty-four barrows (Danbred × Duroc) were randomly allotted to four diets (supplemented with 10 g/kg of crude fibre and 120 mg Zn/kg) that consisted of cellulose and either zinc sulphate (C) or zinc glycinate (ZnGly), or contained potato fibre supplemented with ZnSO_4_ (PF) or ZnGly (PF + ZnGly). Feeding PF can influence the Zn absorption in the small intestine due to reduced zinc transporters MT1 and ZnT1 in the jejunum. The activity of antioxidant enzyme SOD and liver ZnT1, and duodenal iron concentrations were increased in the PF treatments. Dietary ZnGly did not significantly influence the Zn distribution, but it may alter the absorption of Fe and Mn. Given the elevated content of thiol groups and the Zn/Cu ratio in plasma, as well as the altered SOD activity and MT content in the tissues, we can conclude that feeding PF and ZnGly can influence the mineral and antioxidant status of growing piglets. However, further research is needed in order to elucidate the effect of both dietary sources on the transport systems of other minerals in enterocytes.

## 1. Introduction

Trace mineral requirements increase in young piglets, mainly after weaning. Upon weaning, the absorptive capacity of a piglet’s intestine is reduced due to several principal factors, such as changes in the villus structure, an inadequate ability to secrete acid and digestive enzyme levels [1]. Therefore, the diets of early weaning pigs should provide a higher content of trace minerals and mainly highly digestive feed components, while taking their limited feed intake into consideration. Moreover, the immunity or resistance to disease of piglets at this time are additional problems. Zinc, as the second most abundant essential trace element, performs multifarious physiological roles and, as a component of many metalloenzymes, is involved in almost every metabolic pathway of the body [2]. Zinc deficiency may result in a reduced feed intake and growth of pigs, parakeratosis, alopecia, thymus atrophy and impaired immune functions [3].

Zinc recommendations for piglets (<11–25 kg body weight) are 140–150 mg Zn/kg in a complete diet and this decreases with increasing body weight [4]. It is well-known that the composition of feedstuffs and the ratio of dietary nutrients are important factors that affect mineral absorption from the gastrointestinal tract (GIT) of growing piglets in order to maintain or improve their body’s mineral status. Inorganic zinc sulphate widely used for the supplementation of animal feedstuffs is rapidly dissociated to free ions during digestion in the GIT and may interact with other dietary molecules, resulting in mineral losses prior to absorption in the small intestine [5]. For this reason, the majority of dietary zinc is excreted in animal faeces, and after soil fertilisation with manure, the accumulation of zinc is constantly increasing in the soil [6]. Therefore, it is necessary to improve the efficiency and absorption of trace elements by feed composition in order to decrease Zn supplementation in animal nutrition with respect to a substantial reduction of environmental pollution from animal husbandry.

Using mineral amino acid chelates as feed additives in pig nutrition could increase mineral bioavailability and enhance mineral tissue storage. The stable structures of mineral chelates prevent undesirable interactions with feed compounds in the digestive tract, and using active transport pathways via metallotransporters across enterocytes contributes to better mineral absorption and utilisation in the animal body [5,7,8]. Moreover, the elimination of mineral interference with some dietary nutrients, such as phytates or fibres, could improve the mineral efficacy. Therefore, the present study focused on improving the absorption of zinc (Zn) and other trace elements such as iron (Fe), copper (Cu), and manganese (Mn), from the small intestine of piglets using more efficient dietary sources, namely zinc glycine chelate and potato fibre.

The main objective of our study was to investigate the effect of dietary zinc glycinate and potato fibre on trace element absorption and tissue distribution (Zn, Cu, Fe, and Mn) and the activity of Zn-containing enzymes in plasma and various tissues, with a special emphasis on the small intestine of piglets. Since zinc transporter 1 (ZnT1, SLC301A) and metallothionein 1 (MT1) are responsible for the amount of Zn released into the portal circulation for systemic distribution, we investigated the concentrations of these Zn transporters in the small intestine segments of piglets. Our study could elucidate the distribution of Zn in intestine cells and other tissues after feeding with different Zn and fibre sources.

## 2. Materials and Methods

All procedures were done in accordance with European Community guidelines (EU Directive 2010/63/EU for animal experiments), and the experimental protocol was approved by the Local Animal Experimentation Ethics Committee (resolution number WAW2_21/2016, Warsaw University of Life Sciences-SGGW, Warsaw, Poland) and Polish Law on Animal Protection.

### 2.1. Experimental Design, Animals and Diets

A total of twenty-four Danbred × Duroc barrows 40 days old (initial body weight 10.8 ± 0.8 kg) were allocated to one of four dietary treatments, with six replicates per treatment in a completely randomized design. During the feeding period, which lasted for 4 weeks, the piglets were given a cereal-based diet supplemented with 10 g/kg crude fibre and 120 mg Zn/kg from two fibre and zinc sources. The experimental diets consisted of cellulose (Lonocel, Cargill Poland Ltd., Kiszkowo, Poland; 637 g/kg crude fibre) as the fibre source and zinc sulphate (C; ZnSO_4_.H_2_O, reagent grade, Sigma-Aldrich, USA) or zinc chelate of glycine hydrate (ZnGly; Glycinoplex-Zn, Phytobiotics Futterzusatzstoffe GmbH, Eltville, Germany), and potato fibre (Potex, Lyckeby Starch, Kristianstad, Sweden; 197 g/kg crude fibre) with ZnSO_4_ (PF) or zinc glycinate (PF + ZnGly). The diets were isonitrogenous, isoenergetic and prepared to contain a total of 150 mg Zn/kg of complete diet and did not exceed 50 g/kg of total crude fibre content. The diet composition of the basal cereal-based diet, containing either cellulose (for C and ZnGly treatments) or potato fibre (for PF and PF + ZnGly treatments) and a zinc-free vitamin-mineral mix (DSM Nutritional Products Ltd., Mszczonów, Poland), are shown in Table 1.

Piglets were housed individually in pens and kept in a 12 h light/12 h dark regimen. The housing temperature was maintained at 25 °C. Throughout the experiment, the animals had free access to feed and fresh potable water. Body weight and feed intake were measured weekly.

### 2.2. Data Collection

At the end of the feeding period (28 days), the piglets were stunned by electric shock and exsanguinated, followed by tissue collection. Blood samples were collected in heparinised tubes from each piglet and were centrifuged at 3350 g for 10 min. Tissue samples were collected from the liver, pancreas, left kidney cortex, the skeletal muscles m. longissimus dorsi, small intestinal segments and the metacarpal III and IV bones. Sampling was done at identical locations of each relevant tissue. Pieces 2–3 cm long of cut intestine segment samples were washed with saline solution and immediately frozen in liquid nitrogen for alkaline phosphatase (ALP) analysis. For the analyses of the mineral and transporter content or enzyme activity, excised intestinal sections of duodenum, jejunum and ileum were thoroughly rinsed with saline solution, and the intestinal mucosa of each region was then scraped off with sterile glass slides on ice. All tissues and plasma samples were stored at −80 °C until further analysis.

### 2.3. Analytical Methods

The diets were analysed for dry matter, crude protein, crude ash, crude fat, crude fibre, acid detergent fibre expressed exclusive of residual ash (ADFom), starch and phosphorus according to AOAC procedures [9], with values of 934.01, 984.13, 942.05, 920.39, 962.09, 973.18, 996.11, and 965.17, respectively. Lignin (sa) content was determined by the direct sulphuric acid method, as described by Robertson and Van Soest [10]. Neutral detergent fibre (aNDFom) was analysed with a heat stable amylase and expressed exclusive of residual ash, according to Mertens [11]. Hemicellulose content was calculated as the difference between aNDFom and ADFom, whereas cellulose was the difference between ADFom and lignin (sa). Phytic phosphorus was analysed after phytate precipitation with FeCl_3_ and subsequent colorimetric measurement of phosphorus according to AOAC [9] procedure 965.17. Sugar content was analysed using the anthrone method [12] after protein precipitation with Carrez solutions (K_4_Fe(CN)_6_·3H_2_O and Zn(CH_3_COO)_2_·2H_2_O). 

The Zn, Cu, Fe, Ca and Mn concentrations in the diets, plasma and tissues were analysed using a double-beam atomic absorption spectrophotometer (AA-7000 Series, Shimadzu Co., Kyoto, Japan) with a graphite furnace (GFA-7000, Shimadzu Co., Kyoto, Japan). The microwave-assisted digestion method using closed pressure vessels in an MWS 4 Speedwave device (Berghof Co., Germany) was used for the decomposition of all samples in two replicates. All samples, except blood, were wet acid-digested with a concentrated nitric acid and hydrogen peroxide mixture. Plasma Mn concentration and Cu content in muscle were determined using a graphite furnace atomic absorption spectrophotometer with deuterium background correction and pyrolytic-coated graphite tubes [13]. The certificate reference materials of bovine liver BCR-185R, bovine muscle ERM-BB184 and pig kidney ERM-BB186 from the Institute for Reference Materials and Measurements (IRMM, Geel, Belgium), bone ash SRM 1400 (NIST, National Institute of Standards and Technology, Gaithersburg, MD, USA) and ClinCheck control of lyophilized human plasma (Recipe, Munich, Germany) were included in each analysis to verify the instrument accuracy. Mineral concentrations in all tissue samples were expressed as mg/kg of DM, except bone (mg/kg of bone ash weight) and plasma (µg/L, mg/L).

The total superoxide dismutase activity (SOD) was assayed by a modified spectrophotometric method, based on the ability of the enzyme to inhibit pyrogallol autoxidation by 50% [14]. Tissue samples were homogenized in ice-cold Tris and sucrose buffer (10 mM Tris, 0.25 M sucrose, pH 7.4). The homogenates were centrifuged at 10,000× *g* for 30 min at 4 °C, and the supernatants were used for the analysis. The specific Cu/Zn SOD inhibition by potassium cyanide allows the determination of enzyme activity under the same conditions. Enzyme activity is expressed as units of enzyme per mg of tissue protein. The protein concentration was measured using the spectrophotometric method [15]. MT1 and ZnT1 tissue content were determined with the colorimetric method using commercial ELISA kits (Porcine Zn-MT ELISA kit, NeoScientific, London, UK; SLC30A10 MyBioSource San Diego, CA, USA; respectively). 

The analysis of intestinal alkaline phosphatase activity (ALP) in the intestinal sections was performed using a modified simultaneous azo coupling method [16]. The histochemically stained slides were visualised using an Olympus microscope (BX 51) and a digital compact camera (Olympus DP 50) connected to a host computer. Image analysis was performed in the Ellipse program (ViDiTo, Slovakia), where the grey level of each pixel was given a value in the 0–255 range. A Vickers M85a microdensitometer was used to define the standard density values of enzyme activities at a wavelength of 520 nm, which was required for calibration. A special semi-interactive algorithm was used to find relevant pixels along the villus length, and the density of such pixels was measured [17]. The quantification of enzyme activity (pixel intensities) was carried out along the villus length in a whole section of at least four duodenal and jejunal slides, and the mean values recorded referred to one animal.

The enzymatic activity of plasma ALP was evaluated photometrically in a kinetic way using a commercial kit (ALP MEG 500, Erba Lachema, Czech Republic). For photometrical determination of plasma albumin, an assay kit (ALB 500, Erba Lachema, Czech Republic) was used. The amount of thiobarbituric acid reactive substance (TBARS) was measured in blood plasma according to the method of Rice-Evans et al. [18].

The total concentration of thiol groups (TSH) was performed spectrophotometrically at 412 nm, as described by Ellman [19]. Briefly, aliquots of intestine mucosa (0.5 g) were homogenized in buffer (Tris 50 mM, pH 7.4) and subsequently centrifuged at 3000× *g* for 20 min at 4 °C. To determine non-protein thiols (NPSH), the protein fraction was precipitated with 10% trichloroacetic acid, followed by centrifugation, and the supernatant was used for analysis. The colorimetric assay was carried out using Ellman’s reagent and 0.1 M phosphate buffer (pH 7.4). A standard curve using glutathione was constructed in order to calculate the thiol content in the plasma and tissue samples.

The pro-oxidant-antioxidant balance (PAB) in blood serum was analysed according to Koliakos and Hamidi Alamdari [20]. Briefly, 10 µL of serum was mixed in a well of a 96-well microplate with 200 µL of solution containing 3,3’,5,5’ tetramethylbenzidine (TMB), TMB cation, and 5 U of horseradish peroxidase in 0.05 mM phosphate-citrate buffer, pH 5.0. After 12 min of incubation, 100 µL of 2 M HCl was added and incubated for 45 min at room temperature in the dark. The absorbance was measured at 450 nm, with a reference wavelength of 620 nm, using a Maxmat PL multidiagnostic platform (Erba Diagnostics France SARL, Montpellier, France). The balance values were calculated from a standard curve prepared using 1 mM H_2_O_2_, as a representative of the oxidants, and 6 mM uric acid, as a representative of the antioxidants. The standard solutions were mixed in varying proportions, from 100% of 6 mM uric acid to 100% of 1 mM H_2_O_2_. The results were expressed in arbitrary Hamidi-Koliakos (HK). The HK unit is the percentage of H_2_O_2_ in the standard solution multiplied by 6. Therefore, greater HK values indicate higher oxidant concentrations in a sample.

### 2.4. Statistical Analysis

Treatment effects on mineral tissue concentrations, enzyme activities and Zn transporter content were analysed according to a completely randomised 2 × 2 factorial design, with a factorial arrangement of treatments using two-way ANOVA, followed by a Bonferroni post-hoc test for pair-wise comparisons, where appropriate. The statistical model included the treatment effects (fibre and zinc source) and their interaction. When the interaction was significant, Fisher’s Least Significant Difference test (Fisher’s LSD) was applied post hoc to determine significant differences among the means. Pearson correlation was used to describe the relationships between the data sets. All statistical analyses were performed by the GraphPad Prism statistical software (GraphPad Prism version 8.1.2., GraphPad Software, San Diego, CA, USA). Differences between the mean values of the different dietary treatments were considered statistically significant at *p* < 0.05. Data are expressed as means and pooled standard errors of the mean (SEM).

## 3. Results

The concentrations of Zn transporters MT1 and ZnT1 in the liver and small intestine sections of piglets are expressed in µg per gram of wet tissue (Table 2). The concentrations of both MT1 and ZnT1 in jejunum and liver tissues were affected by the fibre source, with significantly increased levels of jejunum in pigs from the dietary treatments containing cellulose fibre (*p* < 0.01 for MT1; *p* < 0.001 for ZnT1), although ZnT1 levels were elevated in the liver of piglets fed both PF diets (*p* < 0.01). Feeding diets with ZnGly increased the ileal ZnT1 content compared with feeding ZnSO_4_ (*p* < 0.05). The content of total and non-protein sulfhydryl groups in the liver and intestinal tissues (expressed as µmol/g tissue) was not affected by the dietary treatment (Table 2).

Regardless of the dietary source, the highest Zn concentration was observed in duodenal and ileal mucosa (85.5 ± 1.2 mg Zn/kg DM; 81.9 ± 1.22 mg Zn/kg DM, respectively) compared to Zn jejunal content (67.3 ± 0.66 mg Zn/kg DM, *p* < 0.0001). An increased ZnT1 content was observed in the duodenal and jejunal mucosa of all piglets (24.9 ± 0.74, 25.3 ± 0.71µg/g, respectively) in comparison with ZnT1 in the ileum (18.8 ± 0.57, *p* < 0.0001). A low MT1 content was recorded in the duodenum (13.2 ± 0.39 µg/g, *p* < 0.001), followed by an increased value in the ileum (18.4 ± 0.65 µg/g, *p* < 0.01), and the greatest MT1 content was in the jejunum (20.4 ± 0.24 µg/g, *p* < 0.01).

Mineral concentrations of Zn, Cu, Fe and Mn in piglet liver, kidney, pancreas, bone, muscle, duodenum, jejunum and ileum tissues are expressed in mg/kg of tissue dry matter (Table 3). Intake of the diets containing potato fibre increased Zn concentrations in the ileal mucosa (*p* < 0.05). A Zn × fibre source interaction was observed for the Zn concentration in jejunal mucosa, with increased Zn levels in the control treatment supplemented with cellulose and ZnSO_4_ compared to the ZnGly or PF treatment (*p* < 0.05). The Zn concentration in the other tissues and plasma of the piglets was not affected by the dietary treatment (Table 3 and Table 4).

Intake of the diets supplemented with ZnGly increased Fe concentrations in the kidney (*p* < 0.05), but the Fe levels in the ileal mucosa were decreased in the treatments (*p* < 0.01). A nearly two-fold increase in Fe concentrations was determined in the duodenal mucosa of both PF treatments (*p* < 0.01). The liver tissue of piglets fed the PF diets showed a tendency towards increased Fe and Cu concentrations (both minerals, *p* = 0.08). Supplementation with the organic Zn source increased Mn concentrations in the jejunal mucosa of piglets (*p* < 0.05). A Zn × fibre source interaction was recorded for the Mn concentration in the kidney, with the highest Mn levels in the PF + ZnGly treatment (*p* < 0.05). Other tissue mineral concentrations were not changed by the dietary treatment (Table 3).

The effect of fibre source was observed in the plasma Cu concentration and plasma ALP activity (*p* < 0.001, *p* < 0.05; respectively); however, a Zn × fibre interaction was also found (Table 4). Increased plasma ALP was found in the control treatment compared to the PF treatments (*p* < 0.05). Intake of the PF diets decreased Cu levels in the plasma, with the lowest concentration in the PF + ZnGly (*p* < 0.001) combination compared to all other treatments. However, plasma Zn levels were unaffected, and decreased plasma Cu levels mainly in the PF + ZnGly treatment resulted in an elevated Zn/Cu ratio, with the highest significant value in this treatment. Moreover, the Zn/Cu ratio was affected by both the zinc and fibre source (both *p* < 0.01), and a Zn × fibre interaction was also recorded (*p* < 0.01). The effect of both dietary sources, as well as a Zn × fibre interaction, was recorded in the plasma levels of non-protein thiols, with lower values of both total and non-protein thiols in the PF treatment (*p* < 0.05). The effect of the Zn source was observed in the plasma albumin content, with increased values in the ZnGly treatments (*p* < 0.057). The concentrations of MT1, TBARS and PAB in plasma were not affected by the dietary treatment (Table 4).

Increased activity of total SOD and Cu/Zn SOD was only recorded in the liver tissue of both PF treatments (*p* < 0.05), while a Zn × fibre source interaction for enzyme activity was determined in the duodenum (Table 5). The highest duodenal activity of total SOD was found in the ZnGly treatment compared to the control and PF + ZnGly treatment (*p* < 0.05). The activity of both SODs did not change in the kidney, pancreas and jejunum, nor did ALP activity in the duodenum, jejunum or liver tissue (data not shown).

### Correlation Analyses

Relationships between Zn transporters, trace elements and other parameters in animal tissues are presented in Table 6. Correlation analyses showed a significant negative correlation between ZnT1 and MT1 levels in the duodenum (r = −0.417; *p* = 0.04), while jejunal ZnT1 positively correlated with MT1 in the jejunum (r = 0.454, *p* = 0.03). MT1 levels in the duodenum negatively correlated with Cu content in the segment of the small intestine (r = −0.517, *p* = 0.011), while a positive correlation was determined between Cu content and MT levels in the jejunum (r = 0.410, *p* < 0.05). No significant correlation was found between the zinc transporters (MT1, ZnT1) and Cu content in the ileum. Correlation analyses showed a positive correlation between Zn tissue concentrations, with the correlation coefficients (r) between kidney Zn and Zn concentrations in the liver, pancreas, jejunum and Cu content in the kidney at 0.577 (*p* = 0.003), 0.405 (*p* < 0.05), 0.486 (*p* = 0.02), and 0.472 (*p* = 0.03), respectively. Zn duodenal content positively correlated with Cu content in the duodenum (r = 0.52, *p* = 0.011), and Zn concentration in the jejunum and ileum (r = 0.43, *p* = 0.036; r = 0.446, and *p* = 0.029, respectively). Ileal Zn content strongly positively correlated with Cu and Mn levels in the ileum (r = 0.625, *p* = 0.002; r = 0.573, and *p* = 0.003, respectively). Cu content in the duodenum positively correlated with duodenal Fe content (r = 0.621, *p* = 0.002). Mn concentration in the kidney positively correlated with Fe kidney content (r = 0.424, *p* = 0.039). Zn concentrations in the pancreas correlated positively with Zn content in the liver (r = 0.551, *p* = 0.005), in each segment of the small intestine (duodenum r = 0.562, *p* = 0.004; jejunum r = 0.629, *p* = 0.001; ileum r = 0.499, *p* = 0.013) and in Zn bone (r = 0.416, *p* < 0.05). A negative correlation was recorded between Zn muscle content and Zn concentrations in bone (r = −0.658, *p* < 0.001), and also in the Zn pancreas, duodenum and ileum (r = −0.474, *p* = 0.022; r = −0.447, *p* = 0.032; r = −0.474, *p* = 0.022, respectively). A positive correlation was recorded between Zn concentrations and ALP activity in the liver (r = 0.506, *p* = 0.012), and plasma ALP and liver ALP activity (r = 0.619, *p* = 0.002). Cu content in the liver positively correlated with Cu/Zn SOD activity in the tissue (r = 0.446, *p* = 0.043).

## 4. Discussion

The zinc active absorption efficiency depends on a number of proteins involved in intestinal Zn metabolism, such as the divalent metal transporter 1 (DMT1), zinc transporter ZIP4, MT1 and ZnT1 [21]. Regulation of Zn absorption or uptake is achieved by the transporter ZIP4, situated in the apical membrane of intestinal epithelial cells, which transports Zn ions into the cytoplasm, though it has been suggested that Zn absorption from ZnGly only partly depends on ZIP4 [22]. The divalent metal transporter DMT1, which is the principal iron transporter with a lower affinity to uptake other divalent metals into enterocytes, is also in the apical membrane; however, zinc regulates DMT1 expression and function [23]. The zinc transporter ZnT1, which decreases intracellular zinc levels due to the efflux of Zn from GIT cells into circulation, is localised at the opposite basolateral membrane of enterocytes, and in this way correlates with the intracellular free Zn^2+^ content. Therefore, ZnT1 is considered to be an intracellular zinc status indicator [21]. An intracellular zinc-binding protein, metallothionein MT1, serves as a dynamic Zn donor and acceptor and an exchangeable Zn pool and demonstrates the tissue distribution of endogenous zinc [24]. MT plays a primary role in Zn homeostasis and the scavenging of reactive oxygen species [21,24]. Given the literature data suggesting that mRNA expression of DMT1 and ZIP4 is susceptible to a low Zn intake, while ZnT1 and MT1 act as key regulators of Zn homeostasis under normal or high Zn conditions [25], and that the diverse impact of ZnGly on the gene expression of zinc absorption-related transporters has been indicated [22], we focused on the transporters ZnT1 and MT1 in the small intestine segments of piglets responsible for the amount of Zn released into portal circulation for systemic distribution.

Zinc, like other trace elements bound in chelates or organic complexes, should be more stable, and this should prevent Zn ions from chelating by phytates or other feed compounds in the GIT, while the better absorption of organically bound Zn is explained by the use of other transport systems [26,27,28]. Organic sources of zinc may make more Zn available to the organism than inorganic zinc supplements, partially due to the stable cyclic structure that makes Zn more easily usable by transcription factor MTF-1, and so MT gene expression could be regulated more effectively [29]. The upregulation of MT1 and ZnT1 mRNA expression was recorded on IPEC-J2 cells treated with different Zn sources, especially in the ZnGly-added treatment [22]. Similarly, organic dietary Zn sources increased gene expression of the zinc transporter ZnT1 in the jejunum of broiler chickens and the apparent Zn retention in piglets [27,30]. In our study, the feeding of both ZnGly diets elevated ZnT1 levels in the ileal mucosa, whereas MT levels and Zn concentration were not affected by the Zn source in any intestinal sections. However, the interaction of a challenge by Zn and also a fibre source was noted for the Zn concentration in the jejunal mucosa. The highest Zn content was noted in the jejunum of pigs fed the diet supplemented with cellulose and ZnSO_4_ compared to the ZnGly or PF diets. Our results indicate that ZnGly did not affect active Zn transport in the duodenum and jejunum, but it seems that it can alter Zn efflux from ileum into circulation without evidence of influencing zinc tissue deposition compared to ZnSO_4_.

Both insoluble and soluble dietary fibres might have unfavourable effects on mineral absorption because of their mineral-binding properties. Cellulose is poorly soluble and fermentable, and the physical entrapment of minerals intrinsic to cellulose due to the formation of insoluble forms and/or large complexes results in decreasing mineral bioavailability [31]. On the other hand, the enhancing effect of soluble/fermentable fibre sources could be related to fermentation products that may increase passive absorption, the lowering of pH or the organic acid production that may have absorption enhancing properties [32]. The fermentation of soluble fibres in the colon often produces short chain fatty acids that can trigger the increased proliferation of epithelial cells, an increased absorptive surface area and hence absorption of some minerals, although this absorption enhancing property does not apply to all minerals and depends on the specific characteristics of the soluble fibre concerned [33]. Based on the mentioned properties of soluble fibres and our previous results indicating a positive effect of dietary potato fibre on mineral digestibility in pigs [34,35], the increased absorption of trace minerals, including zinc from the GIT of piglets, and the better mineral utilisation in tissues, were intended in the PF treatments. The intake of the PF diets decreased the MT1 and ZnT1 concentration in the jejunum of piglets in our study. Intestinal MT regulates Zn absorption and can inhibit dietary Zn uptake by its sequestering capacity [36,37]. Cu has been shown to have a higher affinity than Zn for MT, presumably displacing Zn from MT and reducing Cu absorption [36]. The increased MT levels in the jejunum of piglets fed the cellulose-containing diets depressed the intracellular Zn and Cu concentrations by their sequestration inside the enterocytes bound to MT resulted in an apparent decreased Zn digestibility [38] and decreased Cu concentration in the liver. A tendency towards an increased liver Cu concentration in the PF treatments could result in the higher activity of Cu/Zn-dependent SOD in liver tissue. Liver SOD activity is sensitive to changes in Cu status and the increase in SOD activity is associated with an increased Cu content. Cu may not be safely bound in liver and acts as a pro-oxidant, leading to an increase in Cu/Zn superoxide dismutase activity in the tissue [39,40,41]. Moreover, a positive correlation between Cu/Zn SOD activity and Cu concentration in liver tissue was recorded in our study. 

Regardless of the dietary source of Zn and fibre, the highest contents of ZnT1 and MT1 were observed in the jejunum of piglets, and the Zn concentration decreased significantly in this intestinal section. Higher Zn concentrations were found in the duodenum, followed by an increased ZnT1 content and inversely correlated MT1. A kinetic study on intestinal zinc transport using an intestinal membrane isolated from small intestinal segments of rat indicated the lowest Zn transport rate in the jejunum, while the maximum Zn transport rate in the duodenum, which was not correlated with the mRNA expression of the Zn transporters ZnT1 and ZIP4, but showed an inverse correlation with MTs [42]. The authors suggested that the increased expression of MTs results in a low transport rate of zinc, and the increased relative expression of ZnT1, MT1 and MT2 was only reported in the jejunum compared to other segments of a rat’s small intestine. Our findings suggest a higher Zn absorption rate in the duodenum of piglets, while the lower Zn absorption in the jejunum was probably caused by the intracellular zinc-binding capacity of MTs. The high Zn content in the ileum might be caused by a non-saturable diffusive process, which is a different transport mechanism than the carrier-mediated absorption used in the duodenal and jejunal segments. Similar results were described in small intestinal segments of broilers, where Zn absorption from organic sources was obviously greater than from ZnSO_4_ [43]; however, the intake of ZnGly in our study only increased Zn efflux from the ileum by an increased ZnT1 content.

The correlation analyses regarding Zn tissue concentration showed a strong positive correlation between Zn concentrations in the pancreas and each small intestinal segment, which confirms the important role of the pancreas in the endogenous excretion and reutilisation of zinc, as well as for Zn homeostasis. Zn, Cu and Fe are chemically similar, so the interaction among the elements appears to be tissue-specific and a positive correlation has been found between total Zn and Cu concentrations, mainly in liver and kidney tissues [40,41]. We found a positive correlation between Zn and Cu content in the kidney and ileum. In addition, ileal Zn content was positively correlated with Mn levels and duodenal Cu content was positively correlated with Fe levels in the intestinal segment. The positive correlation between Zn and Cu, Mn, and also Cu and Fe in intestinal tissues may indicate an intracellular trapping of the minerals caused by the impairment of efflux mechanisms. Low intracellular available Cu bound to MT could decrease the efflux of Fe and Mn during their transport out of the enterocytes through the Cu-dependent protein hephaestin that co-operates with their common transporter, ferroportin [44]. Unfortunately, we did not focus on other metallotransporters, so our results cannot clearly indicate any significant competition among the minerals that share the same transporter systems at the level of membrane transport. The effect of the Zn source was found in the increased kidney and reduced ileal Fe content, and the strong positive correlation between liver Fe and ZnT1 contents, mainly in the ZnGly treatments. Intake of the PF diets increased Fe concentrations in the duodenum, which could have led to better Fe utilisation by the liver tissue in the PF treatments. On the other hand, increased duodenal Fe could be caused by impaired Fe efflux out of the eneterocytes. Anyway, further investigation is needed to fully understand the effect of different Zn and fibre sources on Fe transporter mechanisms in enterocytes.

Our results showed a correlation between plasma Zn levels and total thiols or plasma albumin, with the highest values in the plasma of the piglets fed the diet enriched with ZnGly and PF. Plasma thiols were comprised of protein-bound and non-protein sulfhydryl groups, which contribute to the antioxidant capacity of plasma [45]. The most abundant plasma protein is albumin, which is the principal zinc carrier in plasma. The albumin molecule has 17 disulfide bridges and a single free cysteine residue, and given its structure, it is also a powerful radical scavenger in plasma and provides >50% of the total antioxidant of normal plasma [46]. It has been shown that during oxidative stress, plasma Zn levels and the plasma albumin-bound fraction of Zn are decreased, while plasma Cu levels are elevated [47]. Given the highest decrease of plasma Cu levels in the PF + ZnGly treatment, an elevated Zn/Cu ratio was recorded, whereas plasma Zn concentrations were similar in all piglets in our study. Low plasma Cu levels and low Cu/Zn ratios have been noted after excessive Zn treatments due to depressed Cu absorption and increased Cu sequestration in the mucosal cell bound to MT [48,49,50]. Nevertheless, Cu absorption is depressed when Zn is given in high excess over Cu [51], and many changes of the Zn/Cu imbalance could be used as a better indicator of metabolic disturbance than Zn or Cu status alone [52].

The zinc-related enzyme ALP is important in the detoxification of pathogenic bacteria endotoxins and enzyme alterations and can affect the absorptive and secretory ability of the small intestine. During the post-weaning phase, the downregulation of intestinal ALP has been suggested due to inflammatory processes and microbiota imbalance of the intestine of piglets [53]. Suitable feed supplementation can increase intestinal ALP expression, together with modulation of the gastrointestinal microbiota of pigs, and reduce intestinal inflammation or prevent diarrhoea. Although intestinal and liver ALP activities were unchanged by dietary treatments in our study, we noted the altered ALP activity in plasma that positively correlated with liver ALP activity. A positive correlation between Zn content and ALP activity in the liver indicates the importance of Zn in ALP enzymatic activity. However, total serum ALP originates from various tissues, mainly from the liver and bone, as well as from the intestine, spleen or kidney, but the bone-specific ALP isoenzyme predominates in the serum of young growing animals. It has been shown that Zn influences bone formation and mineralisation, and also stimulates ALP activity in osteoblast cells [54]. A recent study showed that the partial replacing of dietary inorganic minerals, including Zn with glycine chelates, increased immature collagen in the bone tissue of pheasants [55]. We can only speculate whether the variability of total plasma ALP could imply different bone formation in our piglets because of unchanged ALP levels in the liver or intestine; however, we did not measure any parameters related to bone formation or mineralisation.

## 5. Conclusions

In conclusion, the present study showed that the dietary intake of potato fibre can influence the absorption of zinc and other trace elements from the small intestine due to affecting the Zn-transporter levels in the jejunum of piglets and increased zinc content in the ileum, which is the main site of non-saturable transport pathways. Feeding the potato fibre diets increased the ZnT1 content in the liver, while the content of both zinc transporters ZnT1 and MT1 was decreased in the jejunum. Similarly, dietary PF altered the activity of the Zn-containing enzymes in the liver SOD and plasma ALP. The substitution of dietary zinc glycine chelate in place of the commonly used zinc sulphate did not significantly influence the Zn distribution, but it may alter the absorption and utilisation of iron and manganese. Both dietary sources affected Fe concentrations in the small intestine and kidney tissues, and elevated the content of total thiols and the Zn/Cu ratio in plasma. We can conclude that feeding potato fibre and Zn glycinate can influence the mineral and antioxidant status of growing piglets, but further research is needed in order to elucidate the effect of both dietary sources on the transport systems of other minerals in enterocytes.

## Figures and Tables

**Table 1:** Ingredients and chemical composition of the cereal-based diets (g/kg).

**Ingredients, g/kg**	**C**	**PF**
Barley	200	200
Wheat	450	450
Soybean meal	170	170
Yellow lupin	60	60
Rapeseed oil	25.5	25.5
Corn starch	33	–
Cellulose	17	–
Potato fibre	–	50
Mineral-vitamin mix ^1^	4	4
Monocalcium phosphate	7	7
Calcium carbonate	19	19
Sodium chloride	3	3
L-Lysine	6	6
DL-Methionine	2	2
L-Threonine	3	3
L-Tryptophan	0.5	0.5
**Analysed Composition ^2^, g/kg**	**C**	**PF**
Dry matter	889	890
Crude ash	47	49
Crude protein	182	183
Crude fat	42	41
Crude fibre	41	40
Sugars	41	39
Starch	378	379
Calcium	9.6	9.5
Total phosphorus	5.1	5.1
Phytic phosphorus	1.9	1.7
aNDFom	108	112
ADFom	55	56
Lignin (sa)	11	10
Cellulose	43	47
Hemicellulose	53	55
Zinc (mg/kg)	142	141
Manganese (mg/kg)	99	94
Copper (mg/kg)	125	132
Iron (mg/kg)	315	300
Gross energy (MJ/kg)	16.8	16.6

C: the diets containing cellulose fibre, PF:the diets containing potato fibre. ^1^ Provided per kg of diet: 14680 IU vitamin A, 1835 IU vitamin D3, 138 mg vitamin E, 4.59 vitamin K3, 2.94 mg vitamin B1, 7.34 mg vitamin B2, 4.40 mg vitamin B6, 44.0 μg vitamin B12, 36.7 mg nicotinic acid, 16.0 mg calcium D-pantothenate, 1.47 mg folic acid, 147 μg biotin, 228 mg Fe, 73.4 mg Mn, 156 mg Cu, 631 μg I, 411 μg Se. ^2^ The chemical composition of the cereal-based diets and mineral content in the feed samples were analysed according to Official Methods of Analysis of AOAC International (AOAC) procedures [9].

**Table 2:** The tissue concentration of the metallothein 1 (MT1), zinc transporter 1 (ZnT1), and total and non-protein-bound thiols (TSH and NPSH, respectively) in piglets fed diets differing in zinc and fibre source.

	Dietary Treatment				SEM	Main Effects		Interaction
	C	ZnGly	PF	PF + ZnGly		Zn	Fibre	Zn × Fibre
**MT1, µg/g tissue**								
Liver	13.1	11.8	11.9	11.5	0.415	0.32	0.41	0.60
Duodenum	12.9	12.5	12.7	14.7	0.451	0.37	0.27	0.19
Jejunum	20.5 ^ab^	21.7 ^b^	16.7 ^a^	19.4 ^a^	0.302	0.36	**0.007**	0.17
Ileum	19.4	19.3	18.9	19.0	0.321	0.97	0.57	0.86
**ZnT1, µg/g tissue**								
Liver	16.7 ^a^	16.7 ^a^	20.0 ^ab^	22.6 ^b^	0.856	0.32	**0.003**	0.34
Duodenum	23.9	27.6	23.7	24.4	0.860	0.21	0.31	0.40
Jejunum	26.2 ^ab^	30.1 ^b^	23.9 ^a^	23.1 ^a^	0.684	0.20	**0.001**	0.06
Ileum	15.9	20.2	18.9	20.3	0.656	**0.02**	0.20	0.22
**TSH, µmol/g tissue**								
Jejunum	5.57	4.89	5.48	5.61	0.140	0.25	0.19	0.10
Ileum	5.62	5.48	5.55	6.12	0.131	0.42	0.29	0.19
**NPSH, µmol/g tissue**								
Jejunum	2.35	1.95	1.78	1.93	0.094	0.49	0.11	0.14
Ileum	1.96	1.89	1.88	2.17	0.068	0.42	0.45	0.19

C: cellulose, ZnGly: zinc chelate of glycine hydrate, PF: potato fibre. ^a,b^ Means within lines with different superscript letters are significantly different (*p* < 0.05) using by Bonferroni’s post hoc test; Bold values denote statistical significance at the *p* < 0.05.

**Table 3:** Tissue Zn, Cu, Fe and Mn concentrations of piglets fed diets differing in zinc and fibre source.

Mineral Concentration	Dietary Treatment				SEM	Main Effects		Interaction
	C	ZnGly	PF	PF + ZnGly		Zn	Fibre	Zn × Fibre
**Zinc, mg/kg DM**								
Liver	425	413	420	392	20.1	0.64	0.76	0.85
Kidney	169	153	150	143	4.60	0.14	0.22	0.60
Pancreas	168	152	160	147	9.51	0.48	0.75	0.93
Bone ^1^	293	287	283	290	3.59	0.96	0.64	0.37
Muscle	43.9	40.3	43.4	43.0	1.37	0.50	0.71	0.57
Duodenum	85.9	85.6	82.9	84.7	1.27	0.79	0.47	0.69
Jejunum	70.1 ^a^	65.5 ^b^	65.7 ^b^	67.8 ^ab^	0.76	0.37	0.45	**0.03**
Ileum	77.2	79.7	84.5	82.1	1.13	0.99	**0.03**	0.25
**Copper, mg/kg DM**								
Liver	90.3	79.3	133.8	92.9	8.21	0.12	0.08	0.36
Kidney	39.3	46.2	41.8	44.6	2.78	0.42	0.95	0.74
Pancreas	2.10	2.10	2.46	2.54	0.173	0.95	0.27	0.89
Bone ^1^	2.67	2.56	2.55	2.27	0.106	0.42	0.38	0.71
Muscle	2.53	2.39	2.62	2.76	0.163	0.99	0.52	0.69
Duodenum	57.3	54.3	75.4	51.6	4.90	0.43	0.18	0.30
Jejunum	12.8	13.5	12.3	10.5	0.515	0.61	0.09	0.22
Ileum	8.47	7.99	7.88	7.13	0.307	0.34	0.26	0.83
**Iron, mg/kg DM**								
Liver	354	342	389	415	15.3	0.83	0.08	0.85
Kidney	140	155	136	152	3.60	**0.04**	0.62	0.91
Pancreas	105	99.0	110	102	3.50	0.21	0.51	0.87
Bone ^1^	167	177	172	175	4.98	0.54	0.92	0.75
Muscle	20.7	22.3	20.8	21.3	0.563	0.36	0.68	0.65
Duodenum	566	719	1329	1187	106	0.50	**0.01**	0.74
Jejunum	36.3	34.9	35.7	38.9	1.16	0.48	0.20	0.09
Ileum	41.0	36.0	41.8	35.4	1.13	**0.01**	0.97	0.75
**Manganese, mg/kg DM**								
Liver	9.92	10.2	9.86	9.51	0.201	0.91	0.39	0.48
Kidney	6.63 ^ab^	6.52 ^ab^	6.20 ^a^	6.94 ^b^	0.119	0.18	0.92	**0.05**
Pancreas	5.06	4.57	4.88	4.57	0.115	0.11	0.72	0.71
Muscle	0.45	0.52	0.52	0.59	0.020	0.09	0.08	0.90
Duodenum	9.95	10.3	8.95	9.54	0.465	0.64	0.38	0.88
Jejunum	4.47	4.98	4.04	4.88	0.156	**0.03**	0.39	0.60
Ileum	3.50	3.58	3.50	3.40	0.128	0.98	0.73	0.75

C: cellulose, ZnGly: zinc chelate of glycine hydrate, PF: potato fibr. ^a,b^ Means within lines with different superscript letters are significantly different (*p* < 0.05) using by Fisher’s Least Significant Difference (LSD) post hoc test. ^1^ Bone mineral concentrations, mg/kg ash weight; Bold values denote statistical significance at the *p* < 0.05.

**Table 4:** Plasma concentration of Zn, Cu, Fe and Mn; total- and non-protein-bound thiols (TSH and NPSH, respectively); albumin (Alb); metalothionein 1 (MT); thiobarbituric acid reactive substances (TBARS); prooxidat-antioxidant balance (PAB); and plasma activity of alkaline phosphatase (ALP) in piglets fed diets differing in zinc and fibre source.

Plasma	Dietary Treatment				SEM	Main Effects		Interaction
	C	ZnGly	PF	PF + ZnGly		Zn	Fibre	Zn × Fibre
Zn, mg/L	0.842	0.877	0.836	0.963	0.025	0.18	0.50	0.44
Cu, mg/L	1.77 ^a^	1.90 ^a^	1.74 ^a^	1.47 ^b^	0.039	0.10	**0.001**	**0.001**
Zn/Cu ratio	0.476 ^a^	0.460 ^a^	0.478 ^a^	0.658 ^b^	0.022	**0.01**	**0.002**	**0.003**
Fe, mg/L	2.79	2.42	2.28	2.53	0.089	0.77	0.36	0.16
Mn, µg/L	5.28	4.93	5.22	5.43	0.220	0.88	0.65	0.57
ALP, U/L	548 ^a^	415 ^ab^	318 ^b^	392 ^ab^	27.97	0.54	**0.02**	**0.04**
Alb, g/L	30.5	32.2	31.7	37.4	1.014	0.06	0.09	0.28
MT, µg/L	13.9	14.1	14.4	13.9	0.110	0.53	0.52	0.15
TSH, mmol/L	0.346 ^A^	0.344 ^A^	0.251 ^B^	0.360 ^A^	0.014	0.06	0.13	**0.03**
NPSH, mmol/L	0.024 ^A^	0.020 ^A^	0.018 ^B^	0.020 ^A^	0.001	**0.05**	**0.04**	**0.04**
TBARS, µmol/L	0.41	0.53	0.45	0.41	0.026	0.36	0.43	0.14
PAB, HK unit	63.3	65.9	81.7	72.9	5.262	0.78	0.26	0.61

C: cellulose, ZnGly: zinc chelate of glycine hydrate, PF: potato fibre. ^a,b^ Means within lines with different superscript letters are significantly different (*p* < 0.05) using by Bonferroni’s post hoc test. ^A,B^ Means within lines with different superscript letters are significantly different (*p* < 0.05) using Fisher’s Least Significant Difference (LSD) post hoc test; Bold values denote statistical significance at the *p* < 0.05.

**Table 5:** Activity of total superoxide dismutase (SOD), Cu/Zn superoxide dismutase (Cu/Zn SOD) and alkaline phosphatase (ALP) in tissues of piglets fed diets differing in zinc and fibre source.

Enzyme Activity	Dietary Treatment				SEM	Main Effects		Interaction
	C	ZnGly	PF	PF + ZnGly		Zn	Fibre	Zn × Fibre
**Total SOD, U/mg protein**								
Liver	24.1	23.5	28.9	25.9	0.782	0.27	**0.05**	0.25
Kidney	7.67	7.91	6.60	8.84	0.428	0.16	0.93	0.25
Pancreas	8.70	6.24	7.94	7.40	0.612	0.25	0.88	0.45
Duodenum	9.93 ^a^	13.5 ^b^	12.7 ^ab^	9.61 ^a^	0.636	0.85	0.64	**0.01**
Jejunum	5.72	7.39	7.13	7.32	0.376	0.24	0.40	0.35
**Cu/Zn SOD, U/mg protein**								
Liver	21.6	21.6	26.3	22.9	0.843	0.25	**0.03**	0.46
Kidney	6.49	6.58	5.50	7.49	0.385	0.19	0.96	0.23
Pancreas	5.73	4.12	4.73	4.20	0.468	0.28	0.64	0.58
Duodenum	5.65	8.13	7.22	5.89	0.457	0.67	0.88	**0.02**
Jejunum	3.44	4.77	3.92	4.49	0.268	0.09	0.86	0.49
**ALP ^1^**								
Duodenum	14.7	14.0	13.6	15.2	0.353	0.36	0.93	0.06
Jejunum	12.2	12.0	12.3	12.8	0.253	0.80	0.44	0.49

C: cellulose, ZnGly: zinc chelate of glycine hydrate, PF: potato fibre. ^a,b^ Means within lines with different superscript letters are significantly different (*p* < 0.05) using Fisher’s Least Significant Difference (LSD) post hoc test. ^1^ The enzyme activity is given as density values (pixel intensities) in duodenal and jejunal enterocytes at a wavelength of 520 nm; Bold values denote statistical significance at the *p* < 0.05.

**Table 6:** Relationships between Zn transporters, trace elements and other parameters in tissues of piglets fed diets differing in zinc and fibre source.

Pearson Correlation		Correlation Coefficient *r*	*p* Value
MT1 duodenum vs.	ZnT1duodenum Cu duodenum	−0.417 −0.517	0.043 0.001
MT1 jejunum vs.	ZnT1 jejunum Cu jejunum	+0.454 +0.410	0.029 0.046
Zn duodenum vs.	Zn jejunum Zn ileum Cu duodenum	+0.430 +0.446 +0.520	0.036 0.029 0.011
Cu duodenum vs.	Fe duodenum	+0.621	0.002
Zn ileum vs.	Cu ileum Mn ileum	+0.625 +0.573	0.002 0.003
Zn kidney vs.	Zn liver Zn pancreas Zn jejunum Cu kidney	+0.577 +0.405 +0.486 +0.472	0.003 0.050 0.016 0.031
Mn kidney vs.	Fe kidney	+0.424	0.039
Zn liver vs.	ALP liver	+0.506	0.012
Cu liver vs.	Cu/Zn SOD liver	+0.446	0.043
Zn pancreas vs.	Zn liver Zn duodenum Zn jejunum Zn ileum Zn bone	+0.551 +0.562 +0.629 +0.499 +0.416	0.005 0.004 0.001 0.013 0.049
Zn muscle vs.	Zn duodenum Zn ileum Zn pancreas Zn bone	−0.447 −0.474 −0.474 −0.658	0.032 0.022 0.022 0.001
Zn plasma vs.	TSH plasma ALB plasma	+0.507 +0.478	0.019 0.024
